# A Chinese Herbal Medicine, Jia-Wei-Xiao-Yao-San, Prevents Dimethylnitrosamine-Induced Hepatic Fibrosis in Rats

**DOI:** 10.1155/2014/217525

**Published:** 2014-06-03

**Authors:** Shu-Chen Chien, Wei-Chiao Chang, Pu-Hua Lin, Wei-Pin Chang, Shih-Chung Hsu, Jung-Chen Chang, Ya-Chieh Wu, Jin-Kuo Pei, Chia-Hsien Lin

**Affiliations:** ^1^Department of Clinical Pharmacy, School of Pharmacy, Taipei Medical University, No. 250, Wuxing Street, Xinyi District, Taipei 110, Taiwan; ^2^Department of Pharmacy, Taipei Medical University Hospital, No. 252, Wuxing Street, Xinyi District, Taipei 110, Taiwan; ^3^Department of Pharmacy, Taipei Medical University-Wan Fang Hospital, No. 111, Section 3, Xinglong Road, Wenshan District, Taipei 116, Taiwan; ^4^Master Program for Clinical Pharmacogenomics and Pharmacoproteomics, School of Pharmacy, Taipei Medical University, No. 250, Wuxing Street, Xinyi District, Taipei 110, Taiwan; ^5^Graduate Institute of Clinical Medicine, College of Medicine, Kaohsiung Medical University, No. 100, Shiquan 1st Road, Sanmin District, Kaohsiung 807, Taiwan; ^6^Cancer Center, Kaohsiung Medical University Hospital, No. 100, Tzyou 1st Road, Kaohsiung 807, Taiwan; ^7^Food and Drug Administration, Ministry of Health and Welfare, No. 161-2, Kunyang Street, Nangang District, Taipei 115, Taiwan; ^8^Department of Healthcare Management, Yuanpei University, No. 306, Yuanpei Street, Hsinchu 300, Taiwan; ^9^Department of Early Childhood Care and Education, Kang-Ning Junior College of Medical Care and Management, No. 137, Lane 75, Section 3, Kangning Road, Neihu District, Taipei 114, Taiwan; ^10^Department of Nursing, School of Medicine, National Taiwan University, No. 1, Section 1, Jen Ai Road, Zhongzheng District, Taipei 100, Taiwan; ^11^Department of Nursing, Ching-Kuo Institute of Management and Health, No. 336, Fu-Hsing Road, Zhongshan District, Keelung 203, Taiwan; ^12^Department of Health Industry Management, School of Health Care Management, Kainan University, No. 1, Kainan Road, Luzhu Shiang, Taoyuan 338, Taiwan

## Abstract

Jia-wei-xiao-yao-san (JWXYS) is a traditional Chinese herbal medicine that is widely used to treat neuropsychological disorders. Only a few of the hepatoprotective effects of JWXYS have been studied. The aim of this study was to investigate the hepatoprotective effects of JWXYS on dimethylnitrosamine- (DMN-) induced chronic hepatitis and hepatic fibrosis in rats and to clarify the mechanism through which JWXYS exerts these effects. After the rats were treated with DMN for 3 weeks, serum glutamic oxaloacetic transaminase (SGOT) and serum glutamic pyruvic transaminase (SGPT) levels were significantly elevated, whereas the albumin level decreased. Although DMN was continually administered, after the 3 doses of JWXYS were orally administered, the SGOT and SGPT levels significantly decreased and the albumin level was significantly elevated. In addition, JWXYS treatment prevented liver fibrosis induced by DMN. JWXYS exhibited superoxide-dismutase-like activity and dose-dependently inhibited DMN-induced lipid peroxidation and xanthine oxidase activity in the liver of rats. Our findings suggest that JWXYS exerts antifibrotic effects against DMN-induced chronic hepatic injury. The possible mechanism is at least partially attributable to the ability of JWXYS to inhibit reactive-oxygen-species-induced membrane lipid peroxidation.

## 1. Introduction

The benefits of Chinese herbal medicines in treating chronic diseases, including chronic liver disease, have recently attracted the attention of Western practitioners. Jia-wei-xiao-yao-san (Kami-shoyo-san; TJ-24, JWXYS) is a traditional complex Chinese herbal medicine consisting of 10 medicinal herbal preparations. JWXYS is an officially approved prescription drug in China and Taiwan. Although some reports have addressed the neuropsychological activities of JWXYS, its hepatoprotective effects have not been adequately clarified [1, 2]. The aim of this study was to investigate the hepatoprotective effects of JWXYS and to evaluate the mechanism through which JWXYS exerts these effects.

To investigate the hepatoprotective effect of JWXYS, chronic liver injury was induced by dimethylnitrosamine (DMN) [3–6]. Studies have reported that DMN can induce lipid peroxidation in the liver, thereby reducing hepatic tissue blood flow and leading to acute liver damage and even fulminant hepatitis [7, 8].

In the present study, we examined the hepatoprotective effect of JWXYS by determining the levels of serum glutamic oxaloacetic transaminase (SGOT), serum glutamic pyruvic transaminase (SGPT), albumin in the serum, and histopathological changes in rat hepatic tissues. The antioxidative effects of JWXYS on liver tissues and the superoxide-dismutase- (SOD-) like activity of JWXYS were evaluated to determine the possible mechanism.

## 2. Methods

### 2.1. Drugs and Chemicals

JWXYS (Kami-shoyo-san; TJ-24) was provided by Koda Pharmaceutical (Taoyuan, Taiwan). It consists of 10 medicinal herb preparations, namely,* Angelica sinensis* radix (3.0 g),* Bupleurum falcatum* radix (3.0 g),* Paeonia albiflora* (3.0 g), glycyrrhizae radix (2.0 g),* Moutan Radicis* cortex (2.0 g),* Gardenia fructus* (2.0 g),* Zingiber officinale* rhizome (1.0 g),* Atractylodis macrocephalae* rhizome (3.0 g),* Poria cocos* (3.0 g), and* Mentha arvensis* (1.0 g). JWXYS was dissolved in 0.9% NaCl at concentrations of 100, 300, and 1000 mg/2 mL before use.

DMN and silymarin were purchased from Sigma (St. Louis, MO, USA). Before use, DMN was dissolved in 0.9% NaCl at a concentration of 1%, and silymarin was dissolved in 1% carboxymethylcellulose at a concentration of 200 mg/2 mL.

### 2.2. Animals

Male Wistar rats (110–130 g; BioLasco Taiwan, Taiwan) were used. The Institutional Review Board at China Medical University (Taichung, Taiwan) reviewed and approved the study protocols. The animals were allowed to acclimate for at least 7 days on a standard laboratory diet (Fu-So, Taipei, Taiwan) under environmentally controlled conditions (25 ± 1°C and 55% ± 5% humidity) with free access to food and tap water. A 12 h light/dark cycle was maintained, and hardwood chips were used as bedding.

In this study, 60 rats weighing 160–240 g were randomly divided into 6 groups: control (normal saline-treated), DMN-treated, DMN-treated + silymarin (200 mg/kg), and DMN-treated + JWXYS (100, 300, and 1000 mg/kg) groups.

### 2.3. Dimethylnitrosamine-Induced Liver Injury and Treatments

Dimethylnitrosamine (DMN) can induce hepatic sinusoidal endothelial injury and coagulation necrosis primarily in the central and periportal regions of the lobule [9, 10]. It is usually used to induce experimental liver fibrosis [4]. In our study, DMN was dissolved in normal saline and then intraperitoneally (i.p.) administered to rats 3 times per week at doses of 10 mg/mL/kg. After 3 weeks of inducing liver damage, DMN was continually i.p. administered to the 5 experimental groups, but not to the normal saline control group (1 mL/kg), for the following 3 weeks. From the beginning of the subsequent 3-week period, JWXYS (100, 300, and 1000 mg/2 mL/kg) was orally administered 3 times per day on 3 days in each week to 3 experimental groups. Before the rats were sacrificed, they were starved for 24 h after the final oral administration of JWXYS. Silymarin (200 mg/2 mL/kg) was orally administered to the silymarin group 3 times per day for 3 weeks.

### 2.4. Serum Biochemical and Pathological Evaluation

The protective effect of JWXYS against DMN-induced liver injury was evaluated by assessing the SGOT, SGPT, and serum albumin levels and by examining histopathological sections of the liver of all experimental animals [7].

### 2.5. Antioxidative Effect of Jia-Wei-Xiao-Yao-San

A study reported that (+)-alpha-tocopherol (vitamin E) can scavenge DMN-induced superoxide-free radical production [11]. Therefore, vitamin E was used as a positive control in this study.

To compare the antioxidative effects of JWXYS and vitamin E (0.5 mM) (as a positive control) in rat liver homogenates, FeCl_2_-induced lipid peroxidation was determined based on the formation of a malonic dialdehyde- (MDA-) thiobarbituric acid (TBA) product according to a modified method described by Yuda et al. [12].

Furthermore, to evaluate the inhibitory activity of JWXYS against DMN-induced lipid peroxidation, MDA-TBA products of JWXYS- (100, 300, and 1000 mg/kg) treated rat livers were measured using the method described by Yuda et al. [12].

### 2.6. Superoxide-Dismutase-Like Activity Test

To evaluate the superoxide-scavenging activity of JWXYS, a cytochrome C reduction method developed by McCord and Fridovich was used [13]. Each data point represents the percent of superoxide inhibition (SI, %), and each assay was conducted in triplicate. Xanthine oxidase (XOD) converts xanthine to uric acid, yielding superoxide anions as a byproduct and subsequently converting ferricytochrome C directly to ferrocytochrome C, which exhibits absorbance at 550 nm. When a compound exhibits superoxide-scavenging activity, a reduction in ferricytochrome C occurs. Inhibition of XOD can reduce the production of superoxide anions, and XOD inhibition was measured according to the method described by Frederiks and Bosch [14].

### 2.7. Statistical Analysis

Statistical significance was calculated by conducting a one-way analysis of variance coupled with Dunnett's test. *P* values less than 0.05 indicated statistical significance.

## 3. Results

### 3.1. Body Weight

As shown in Table 1, after treatment with DMN for 42 days, the body weights of rats significantly decreased (*P* < 0.05). By contrast, the body weights of the rats significantly increased (*P* < 0.05) after they were treated with JWXYS (1000 mg/kg) in the second 3-week period compared with those of the group treated with only DMN.

### 3.2. Histological Observations

**Figure 1:**
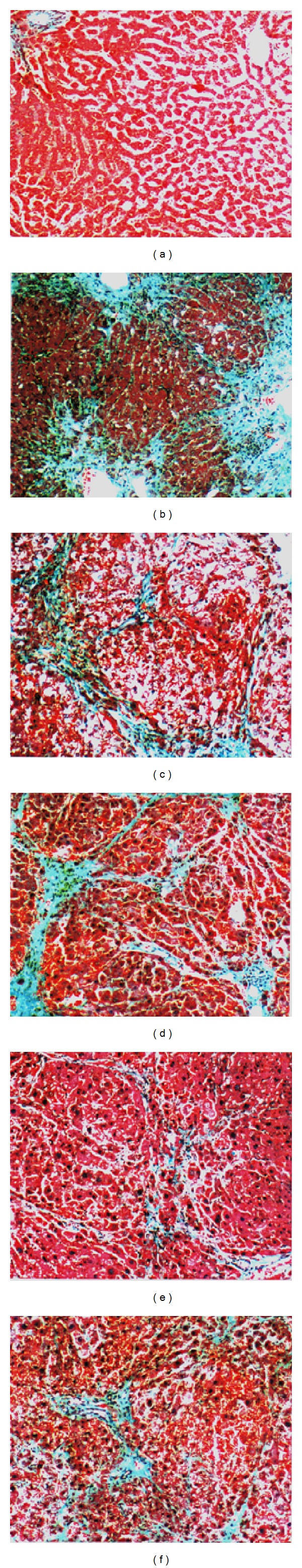
Histological examination performed by conducting Masson trichrome staining, 100x of JWXYS-treated and untreated rat liver. (a) vehicle, normal saline; control, normal liver; (b) DMN alone with marked widening, inflammation, and fibrotic portal tracts with bridging fibrosis. Nodular transformation was apparent. (c) DMN + 200 mg/kg of silymarin with moderate widening and fibrotic portal tracts with occasional bridging fibrosis; (d) DMN + 100 mg/kg of JWXYS with moderate widening and fibrotic portal tracts with bridging fibrosis and mild inflammatory cell infiltration; (e) DMN + 300 mg/kg of JWXYS with focal mild fibrotic change of the portal tracts and bridging fibrosis; (f) DMN + 1000 mg/kg of JWXYS with mild to moderate fibrotic portal tracts with bridging fibrosis.

Histological observations confirmed the hepatoprotective effect of JWXYS.  Figure 1(a) shows a normal control rat liver, in which no necrosis or inflammation was observed. By contrast, the liver of rats treated with 10 mg/kg of DMN exhibited marked widening, inflammation, and a fibrotic portal area, with irregular borders forming a spiked appearance and portal-bridging fibrosis. Nodular transformation was apparent (Figure 1(b)). In the liver of silymarin- (200 mg/kg) treated rats, only moderate widening and a fibrotic portal area with bridging fibrosis were observed (Figure 1(c)).

The protective effect of JWXYS (100, 300, and 1000 mg/kg) against DMN-induced chronic liver injury was determined by examining histological changes in rat livers. The livers of the rats in the JWXYS (100 mg/kg) group exhibited moderate widening and a fibrotic portal area with bridging and mild inflammatory cell infiltration, which were also observed in the silymarin- (200 mg/kg) treated group (Figure 1(d)). By contrast, in the livers of rats treated with 300 and 1000 mg/kg of JWXYS, only mild focal fibrotic changes of the portal area and mild bridging were noted (Figures 1(e) and 1(f)). Thus, histological improvements in the livers of rats treated with 300 and 1000 mg/kg of JWXYS were obviously more substantial than those in the livers of rats treated with 100 mg/kg of JWXYS or 200 mg/kg of silymarin.

### 3.3. Serum Biochemical Assay

As shown in Table 2, the SGOT and SGPT levels of the experimental groups were significantly elevated, whereas the albumin level decreased after DMN treatment compared with those of the normal control group. The results indicated that DMN can induce chronic active hepatitis and diffuse liver injury.

By contrast, treatment with JWXYS (100, 300, and 1000 mg/2 mL/kg) or silymarin (200 mg/2 mL/kg) during the second 3-week period significantly prevented further DMN-induced elevations in serum GOT and GPT levels and a further decrease in the serum albumin level (Table 2). According to these results, JWXYS exhibited strong hepatoprotective effects against DMN-induced chronic hepatic injury in rats. The hepatoprotective ability of 300 and 1000 mg/kg of JWXYS was greater than that of silymarin alone.

### 3.4. FeCl_**2**_-Stimulated Lipid Peroxidation* In Vitro*

Although JWXYS (10 mg/kg) significantly inhibited 51.71% of FeCl_2_-stimulated lipid peroxidation* in vitro*, 0.5 mM vitamin E inhibited 71.10% of FeCl_2_-stimulated lipid peroxidation. JWXYS (0.1, 1.0, and 10 mg/kg) significantly and dose-dependently inhibited FeCl_2_-stimulated lipid peroxidation* in vitro*. Nevertheless, we proved that JWXYS (100, 300, and 1000 mg/kg) dose-dependently inhibited DMN-induced lipid peroxidation in rat livers (*in vivo*). No significant difference was observed between treatments with 1000 mg/kg of JWXYS (69.00%) and 0.69 mM vitamin E (71.16%) (Table 3).

### 3.5. Superoxide-Dismutase-Like Activity

The SOD-like activity (SI%) of JWXYS (0.01, 0.1, and 1.0 mg/mL) was examined to clarify the superoxide-scavenging ability. The activity of vitamin E (8 mg/kg) was set to 100% of SOD-like activity. The results indicated that the SOD-like activity levels of JWXYS at 0.01, 0.1, and 1.0 mg/mL were 33.77%, 68.83%, and 94.81%, respectively. JWXYS at 1.0 mg/mL exhibited the strongest superoxide-scavenging activity (94.81%) (Table 4).

XOD converts xanthine to uric acid, thus yielding superoxide anions as a byproduct [15]. Therefore, inhibition of XOD can reduce the production of superoxide anions. As shown in Table 4, the levels of XOD inhibition (SI%) induced by JWXYS at 0.01, 0.1, and 1.0 mg/mL were 11.90%, 14.29%, and 83.33%, respectively. These results showed that JWXYS dose-dependently inhibited XOD activity.

## 4. Discussion

Chronic hepatitis B, hepatitis C, and alcoholism are the major risk factors for developing liver cirrhosis and hepatocellular carcinoma. The pathway for the progression to liver cirrhosis is the fibrotic process in the liver of patients with the aforementioned risk factors [16, 17]. Therefore, antifibrotic therapy for chronic hepatitis is a crucial topic. A previous study revealed that the combination of interferon and ribavirin exerted an antifibrotic effect in treating chronic hepatitis C. However, the effect was limited, and concerns have been raised regarding the costs and side effects [18–20]. Studies have suggested that alternative agents such as silymarin, sho-saiko-to, halofuginone, imatinib mesylate, phosphodiesterase inhibitors, and endothelin-A-receptor and angiotensin antagonists have antifibrotic effects in chronic hepatitis [20, 21].

Shimizu et al. reported that the traditional Chinese herbal medicine sho-saiko-to (as it is called in Japanese), which features effects similar to those of silibinin, exhibited radical-scavenging ability and antifibrotic properties in activated hepatic stellate cells* in vitro* and in porcine serum-induced fibrosis* in vivo* [22]. Like sho-saiko-to, JWXYS is an option for treating patients with chronic hepatitis in China and other Asian countries.

As shown in Table 1, JWXYS eliminated the effect of DMN-induced body weight loss in rats. In addition, we observed that JWXYS treatment inhibited DMN-induced hepatic injury and fibrosis in a dose-dependent manner. The effects of JWXYS were stronger than that of silymarin (Figures 1(e) and 1(f)). As shown in Table 2, JWXYS significantly diminished the DMN-induced hepatitis and severe diffuse liver injury, thereby disrupting the sequence of events leading to liver fibrosis.

Recent research has indicated that chronic liver injury and hepatic fibrosis are related to oxidative stress, including that caused by reactive oxygen species (ROS) and lipid peroxidation [23, 24]. Studies have suggested that antioxidants such as SOD, catalase, and estradiol significantly prevent lipid peroxidation and exacerbation of liver fibrosis [25–27]. Indeed, as shown in Table 4, JWXYS inhibited XOD activity in a dose-dependent manner. XOD converts xanthine to uric acid, yielding superoxide anions as a byproduct [15], and the inhibition of XOD can substantially reduce the production of superoxide anions, thus reducing superoxide-induced lipid peroxidation and subsequent liver fibrosis.

Furthermore, our study revealed that the Chinese herbal medicine, JWXYS, significantly inhibited FeCl_2_-stimulated (*in vitro*) and DMN-induced (*in vitro*) lipid peroxidation in a dose-dependent manner (Table 3). In the cytochrome C test, we confirmed that JWXYS exhibits a dose-dependent antioxidative activity (Table 4). Furthermore, in another experiment we performed, JWXYS exhibited no hepatotoxic effect in Wistar rats even at a dose of 2000 mg/kg orally administered for 6 weeks (data not shown).

Our study has limitations because, to extrapolate conclusions regarding humans from experimental data on rats, the dose must be validated in clinical trials. The mechanism through which JWXYS acts in cellular pathways remains undetermined. Immunohistochemical analysis of markers such as alpha-smooth muscle actin in activated hepatic stellate cells and sets of* in vitro* culture studies on parenchymal cells and nonparenchymal cells might provide additional evidence supporting the molecular mechanisms through which JWXYS exerts antifibrotic effects. Moreover, in an ongoing follow-up study conducted in our laboratory, JWXYS exhibited strong free-radical-scavenging and antioxidant activity and inhibited free-radical-induced hepatic fibrosis. We will continue to conduct research on this topic.

## 5. Conclusions

We reported that JWXYS exhibits obvious dose-dependent antifibrotic effects against chronic hepatic injury that are similar to those exhibited by sho-saiko-to. This protective effect was noted even in a condition in which DMN was continually administered. The mechanism may at least partially be due to the inhibitory effect of JWXYS on lipid peroxidation and its superoxide-scavenging activities. We hope that, after additional evidence has been accumulated, JWXYS will be applied as an alternative clinical medication for chronic hepatitis and hepatic antifibrotic therapy because of its efficiency, low cost, and fewer side effects.

## Figures and Tables

**Table 1:** Effects of JWXYS and silymarin on the body weights of rats with DMN-induced chronic liver injury.

Group		Time after DMN treatment (day)			
		0	14	28	42
A	Normal control	148.0 ± 12.2	214.0 ± 13.3	282.0 ± 33.9	348.8 ± 35.5
B	DMN (10 mg/kg)	143.7 ± 23.3	190.7 ± 19.3	225.7 ± 10.5	265.7 ± 11.0*
C	DMN (10 mg/kg) + silymarin (200 mg/kg)	145.0 ± 20.0	193.2 ± 16.8	273.2 ± 22.0	325.2 ± 28.7
D	DMN (10 mg/kg) + JWXYS (100 mg/kg)	144.7 ± 17.3	190.7 ± 16.9	272.7 ± 16.3	324.8 ± 16.2
E	DMN (10 mg/kg) + JWXYS (300 mg/kg)	145.0 ± 16.9	190.2 ± 16.9	273.0 ± 16.4	325.6 ± 17.2
F	DMN (10 mg/kg) + JWXYS (1000 mg/kg)	145.0 ± 20.0	195.2 ± 16.9	277.0 ± 17.2	331.8 ± 18.2^#^

Each value is presented as the mean ± SE (*n* = 10).

*P < 0.05, significantly different from Group A.

^#^ *P* < 0.05, significantly different from Group B.

**Table 2:** Effects of JWXYS on the SGOT, SGPT, and albumin levels in rats with DMN-induced chronic liver injury.

Group		SGOT	SGPT	Albumin
		(IU/L)	(IU/L)	(g/dL)
A	Normal control	105.3 ± 13.9	24.2 ± 3.3	4.45 ± 0.02
B	DMN (10 mg/kg)	320.1 ± 42.3^##^	80.3 ± 11.8^##^	3.24 ± 0.04^#^
C	DMN (10 mg/kg) + silymarin (200 mg/kg)	200.4 ± 17.6**	66.7 ± 11.1**	3.87 ± 0.14**
D	DMN (10 mg/kg) + JWXYS (100 mg/kg)	211.3 ± 15.6**	70.1 ± 7.6*	3.76 ± 0.15
E	DMN (10 mg/kg) + JWXYS (300 mg/kg)	193.6 ± 11.8**	63.3 ± 7.3**	3.86 ± 0.16**
F	DMN (10 mg/kg) + JWXYS (1000 mg/kg)	168.3 ± 9.9**	47.3 ± 6.8**	3.96 ± 0.18**

Values are presented as the mean ± SE (*n* = 10).

^#,##^ *P* < 0.05 and 0.01, significantly different from Group A.

^∗,∗∗^ *P* < 0.05 and 0.01, significantly different from Group B.

One-way analysis of variance coupled with Dunnett's test.

*P* values < 0.05 indicated significance.

**Table 3:** Inhibitory effects of various doses of JWXYS on FeCl_2_-induced (*in vitro*) and DMN-induced (*in vivo*) lipid peroxidation in rat livers.

Group		MDA (nmole/mg protein)	Inhibition rate (%)
A	Normal control	1.69 ± 0.11	

*In vitro *			
B	FeCl_2_	2.63 ± 0.08**	
C	FeCl_2_ + vitamin E (0.5 mM)	0.76 ± 0.05^##^	71.10
D	FeCl_2_ + JWXYS (0.1 mg/kg)	2.10 ± 0.05^##^	20.15
E	FeCl_2_ + JWXYS (1.0 mg/kg)	1.44 ± 0.06^##^	45.25
F	FeCl_2_ + JWXYS (10 mg/kg)	1.27 ± 0.03^###^	51.71

*In vivo *			
G	DMN	3.71 ± 0.05**	
H	DMN + vitamin E (0.69 mM)	1.07 ± 0.05^△△△^	71.16
I	DMN + JWXYS (100 mg/kg)	2.79 ± 0.05^△△^	24.80
J	DMN + JWXYS (300 mg/kg)	2.21 ± 0.06^△△^	40.43
K	DMN + JWXYS (1000 mg/kg)	1.15 ± 0.03^△△△^	69.00

Each value is presented as the mean ± SE (*n* = 6). Vitamin E was used as the positive control.

**P < 0.01, significantly different from Group A.

^##,###^ *P* < 0.01 and 0.001, significantly different from Group B.

^△△,△△△^ *P* < 0.01 and 0.001, significantly different from Group G.

One-way analysis of variance coupled with Dunnett's test.

*P* values < 0.05 indicated significance.

**Table 4:** Effects of JWXYS on SOD-like activity and XOD inhibition.

Group		SOD-like activity (SI%)*	XOD inhibition (SI%)^#^
A	Vitamin E (8 mg/kg)	100.00	
B	JWXYS (0.01 mg/mL)	33.77	11.90
C	JWXYS (0.1 mg/mL)	68.83	14.29
D	JWXYS (1.0 mg/mL)	94.81	83.33

*Measured using the cytochrome C reduction test.

^#^Measured using the XOD inhibition test.

Vitamin E was used as the positive control.

SI: superoxide inhibition.

## Abbreviations

DMN: Dimethylnitrosamine
JWXYS: Jia-wei-xiao-yao-san
MDA: Malonic dialdehyde
SGOT: Serum glutamic oxaloacetic transaminase
SGPT: Serum glutamic pyruvic transaminase
SI: Superoxide inhibition
SOD: Superoxide dismutase
TBA: Thiobarbituric acid
XOD: Xanthine oxidase.
